# MMP2 and MMP9 contribute to lung ischemia–reperfusion injury via promoting pyroptosis in mice

**DOI:** 10.1186/s12890-022-02018-7

**Published:** 2022-06-15

**Authors:** Peng Zhou, Nai-Cheng Song, Zhi-Kun Zheng, Yi-Qing Li, Jin-Song Li

**Affiliations:** 1 Department of Vascular Surgery, Union Hospital, Tongji Medical College, Huazhong University of Science and Technology, Wuhan, China; 2 Department of Thoracic Surgery, Union Hospital, Tongji Medical College, Huazhong University of Science and Technology, Wuhan, China

**Keywords:** Lung ischemia–reperfusion injury, Matrix metalloproteinase, Pyroptosis

## Abstract

**Background:**

Lung ischemia–reperfusion injury (LIRI) is a cause of poor prognosis in several lung diseases and after lung transplantation. In LIRI, matrix metalloproteinases and pyroptosis indicators change in parallel, both of them involvement of inflammatory modulation, but it is unclear whether they are related to each other.

**Methods:**

We analyzed the matrix metalloproteinases (MMPs) changes from RNA sequencing (RNA-Seq) data of human transplantation and rat ischemia–reperfusion lung tissues in the Group on Earth Observations (GEO) database. Then established the mouse LIRI model to validate the changes. Further, the severity of lung injury was measured after intervening the matrix metalloproteinases changes with their selective inhibitor during Lung ischemia–reperfusion. Meanwhile, lung, pyroptosis was assessed by assaying the activity of Caspase-1 and interleukin 1β (IL-1β) before and after intervening the matrix metalloproteinases changes.

**Results:**

The RNA-Seq data revealed that matrix metallopeptidase 2 (MMP2), matrix metallopeptidase 9 (MMP9) mRNA expression was elevated both in human lung transplantation and rat lung ischemia–reperfusion tissues, consistent with the change in our mouse model. At the same time, the activity of Caspase-1 and IL-1β were increased after LIRI. While, the lung injury was attenuated for the use of MMP2 and MMP9 selective inhibitor SB-3CT. Likewise, lung pyroptosis alleviated when treatment the mice with SB-3CT in LIRI.

**Conclusion:**

We conclude that MMP2 and MMP9 are involved in the process of LIRI, the mechanism of which is related to the promotion of lung pyroptosis.

**Supplementary Information:**

The online version contains supplementary material available at 10.1186/s12890-022-02018-7.

## Background

Lung ischemia–reperfusion injury (LIRI) occurs under a variety of clinical conditions, such as pulmonary thrombosis, cardiac arrest, lung transplantation, trauma and cardiopulmonary bypass surgery [1–4]. LIRI is a complex pathophysiological process, leading to, such as inflammation, oxidative stress and cell death in the lung [5, 6]. The development of LIRI leads to prolonged duration of mechanical ventilation, prolonged hospital stay, increased medical expenditures and high morbidity and mortality [7, 8]. Therefore, it is of great practical significance to explore the prevention of LIRI.

Pyroptosis is an intrinsic inflammatory process of caspase-1-dependent programmed cell death, which is a newly discovered mode of programmed cell death in addition to apoptosis and necrosis [9, 10]. As we describe in our published paper [11], classic pyroptosis is initiated by caspase-1-dependent inflammasomes such as NLRP3, which is one of the most common examples, which consist of pattern recognition receptors (PRR), apoptosis-associated speck-like proteins containing CARD (ASC), and effector protein caspase-1 precursors (pro-caspase-1). The NLRP3 inflammasome complex can recognize pathogen-associated molecular patterns (PAMPs) or damage-related molecular patterns (DAMPs), and promote pro-caspase-1 activation through self-cleavage into active caspase-1. Activated caspase-1 cleaves the precursors of IL-1β and IL-18 into mature IL-1β and IL-18, which activate the immune response and induce inflammation [12]. Studies show that pyroptosis plays an important role in the development of infectious, atherosclerotic, and neurological-related diseases [13–15]. Our previous study showed that human alveolar epithelial cells co-cultured with monocytes under hypoxia–reoxygenation conditions underwent pyroptosis, suggesting that cell pyroptosis may be involved in LIRI [11]. However, the mechanisms of its function require further elucidation.

During LIRI, degradation of the extracellular matrix, activation and chemotaxis of inflammatory cells, and release of inflammatory factors are closely associated with injury, and MMPs, also known as matrix proteins, may play a central role in these processes [16, 17]. It has been reported that IRI in the kidney, cerebrum, myocardium, and other organs is associated with elevated MMP2 and MMP9 levels [18–22], but the mechanism of their association is unclear. MMP2 and MMP9 play an important role in degrading basement membranes, allowing neutrophil (PMN) migration and promoting inflammation [23, 24]. In many diseases, MMP2, MMP9, and pyroptosis indicators change in parallel, but it is unclear how they are related to each other [25–28]. However, whether an association exists between them in LIRI has rarely been studied.

Therefore, we hypothesized that MMPs is involved in modulating LIRI through regulating pyroptosis. In this study, we established the mouse LIRI model to validate the changes of MMP2 and MMP9, and lung pyroptosis. Furthermore, the severity of lung injury and pyroptosis were measured after intervening the MMP2 and MMP9 changes using of their selective inhibitor, to illustrate the relationship of MMP2/MMP9 and pyroptosis.

## Materials and methods

### Data collection and analysis in the GEO database

We searched the GEO database (http://www.ncbi.nlm.nih.gov/geo/) for the lung transplantation and ischemia–reperfusion mRNA expression datasets and obtained two independent datasets: GSE127003 (n = 92 samples) and GSE9634 (n = 18). GSE127003 dataset is recorded before and after human lung transplantation RNA-Seq data of lung tissue. GSE9634 dataset is recorded the rat lung ischemia–reperfusion tissue RNA-Seq data after IR-30 min and IR-3 h. The GER2R online tool was used to analyze differential gene expression, mainly including MMP2, TIMP2, MMP9, and TIMP1.

### Animals

We purchased 15 male adult BALB/c mice (6–8 weeks old, 25 ± 2 g) from SHULAIBAO Biotech (Hubei, China). The mice were in separate cages in a relatively clean environment and fed a standard diet and had access to water ad libitum. The animal experiments in this study were approved by the hospital ethics committee, and the experimental procedures were conducted in accordance with the unit and national regulations on the management and use of laboratory animals. All animals received care in compliance with the Principles of Animals Use Committee (NIH Publications No. 8023, revised 1978). The project was approved by the Wuhan Union Hospital Ethics Committee (REC 08/H1202/137), China.

### Grouping and models

Mice were randomly divided into three groups: sham-operated group (sham), ischemia–reperfusion group (IR), and SB-3CT pretreatment group (IR-SB). SB-3CT (10 mg/kg, Cat #S7430, Selleck), dissolved in sodium citrate solution, was intraperitoneally injected every other day for 14 days, a widely prescribed MMP2/MMP9 inhibitors in previous studies [29–31]. The experimental procedures in Sham group and IR group were performed as Xu, one of our Lab Members, described [32]. Briefly, mice were anesthetized with 100 mg/kg 1% sodium pentobarbital (Sigma-Aldrich, USA) intraperitoneally (i.p.) and 4.0 mg/kg atropine (Hefeng, Shanghai, China) i.p. The mice received anticoagulation treatment with 500 U/kg heparin sodium (Qianhong, Changzhou, China) i.p 15 min before operation. Endotracheal intubation was performed with a 20G intravenous catheter connected to a small animal ventilator (ALC-V8, Shanghai, China); the tidal volume was adjusted to 3.0 ml room air, the respiratory rate to 120 per minute, and the inspiratory/expiratory ratio to 1:2. After opening the left thoracic cavity between the 3rd and 4th rib, a microvascular clamp was used to clamp the lung hilum. The clamp was removed 1 h later, and the chest cavity was closed. After reperfusion for 2 h, mice were sacrificed for specimen analysis. A sham group received thoracotomy only. Blood, 0.5–0.7 ml, was obtained from the inferior vena cava of fully anesthetized mice during euthanasia, and the left lung was perfused with 2 ml saline, the residual blood was washed out, and the left lung specimen was retained. Each lung was divided into four parts, one to detect protein, one to detect RNA, one to detect MPO activity and one to make tissue sections (5 μm). During the study three mice died (1 from the IR group and 2 from the SB-IR group).

### Hematoxylin and eosin (H&E) staining

The left lung tissue was fixed in 4% paraformaldehyde for 24 h. Paraffin-embedded, 4 μm serial sections were routinely stained with H&E (xylene dewaxing, hematoxylin staining for 5 min, hydrochloric acid ethanol treatment for 30 s, water immersion for 15 min, and eosin staining for 2 min). This was followed by light microscopy observation.

### Histology scoring of lung injury

Lung injury was pathologically scored under the microscope by an experienced pathologist. The criteria followed were as described in the literature: hemorrhage in the alveolar space (1, with hemorrhage; 0, without hemorrhage); the degree of inflammatory cell infiltration (0, normal; 1, minimal change; 2, slight change; 3, moderate change; 4, severe change); pulmonary hyaline membrane formation and interstitial edema (0, normal; 1, minimal change; 2, slight change; 3, moderate change; 4, severe change) [33]. The scores for each criterion were summed to obtain an overall score for each animal.

### Analysis of myeloperoxidase (MPO) activity

The cryopreserved left lung tissue was homogenized with frozen lysis solution and centrifuged. The supernatant was processed by an MPO activity colorimetric assay kit (Cat# SP14386, SPBIO company, Wuhan, China). The samples were measured at 470 nm absorbance (A) for 1 min and calculated as U/mg protein.

### Estimation of wet/dry mass ratio

The wet weight of the left lung tissue was measured with an electronic balance and then dried in an oven at 60 ℃ for 24 h to a constant weight. The dry weight was weighed to obtain the wet-to-dry weight ratio (W/D), which was used to assess the degree of pulmonary edema and pulmonary congestion after IR.

### Real-time quantitative PCR

The RNA extracted from lung tissue was reverse-transcribed into cDNA using a cDNA Synthesis Kit (Cat #RR037A, Takara). Real-time quantitative PCR was performed on an ABI StepOne Plus System (Applied Biosystems, Foster City, CA, USA) using SYBR Premix Ex Taq (Cat# RR420A, Takara). The primers used were as follows: MMP2, 5′-AACGGTCGGGAATACAGCAG-3′ (forward) and 5′-GTAAACAAGGCTTCATGGGGG-3′ (reverse); TIMP2, 5′-TACCGGTTCTGAAAGACGGC-3′ (forward) and 5′-CCCAAAGGTTCGTTTGCTCG-3′ (reverse); MMP9, 5′-GCAGAGGCATACTTGTACCG-3′ (forward) and 5′-TGATGTTATGATGGTCCCACTTG-3′ (reverse); TIMP1, 5′-CGAGACCACCTTATACCAGCG-3′ (forward) and 5′-ATGACTGGGGTGTAGGCGTA-3′ (reverse). Primer sequences were obtained from PrimerBank, all primers were synthesized from Sangon Biotech. The mRNA levels of target genes were normalized to GAPDH using the 2^−△△CT^ method.

### Western blot analysis

The experimental procedures were performed as our published paper described [34]. The extracted proteins were separated using 10% SDS electrophoresis before transfer onto a nitrocellulose membrane. The blots were cut prior to hybridisation with antibodies during blotting. The membrane was separately probed with the respective primary antibody (MMP2, Cat# A11144; MMP9, Cat# A0289, ABclonal; caspase-1, Cat# 22,915–1-AP, Proteintech; and IL-1β, Cat# 16,806–1-AP; Proteintech) for 8 h at 4 ºC followed by incubation with horseradish-peroxidase-labeled secondary antibody for 2 h at 37 ºC. Enhanced chemiluminescence reagent (Cat# MA0186, Meilunbio) was then added to the blots and the bands were analyzed using ImageJ software (NIH, USA).

### Immunohistochemistry

The experimental procedures were performed as Xu, one of our Lab Members, described [32]. Paraffin-embedded lung sections (5 mm) were prepared. Then, sections were incubated with rat anti-caspase-1/IL-1β antibody (1:100 dilution) at 4 °C overnight. Subsequently, the sections were incubated with horseradish peroxidase (HRP)-conjugated secondary antibody for 30 min at room temperature. Fresh 3,3′-diaminobenzidine (DAB) solution was added to the sections, and the sections were counterstained with hematoxylin. Images were captured under microscopy.

### Statistical analysis

The statistical procedures were performed as our published paper described [11]. Date were given as the mean ± SEM or median (interquartile range) wherever appropriate. Normally and abnormally distributed quantitative variables were compared using the Student’s *t*-test and the Mann–Whitney U test, respectively. The least significant method (LSD method) in one-way ANOVA was used for pairwise comparisons between different groups. Values of P < 0.05 were considered statistically significant.

## Results

### Both before and after human transplantation and rat ischemia–reperfusion lung tissues show increased mRNA expression of MMP2 and MMP9

**Fig. 1:**
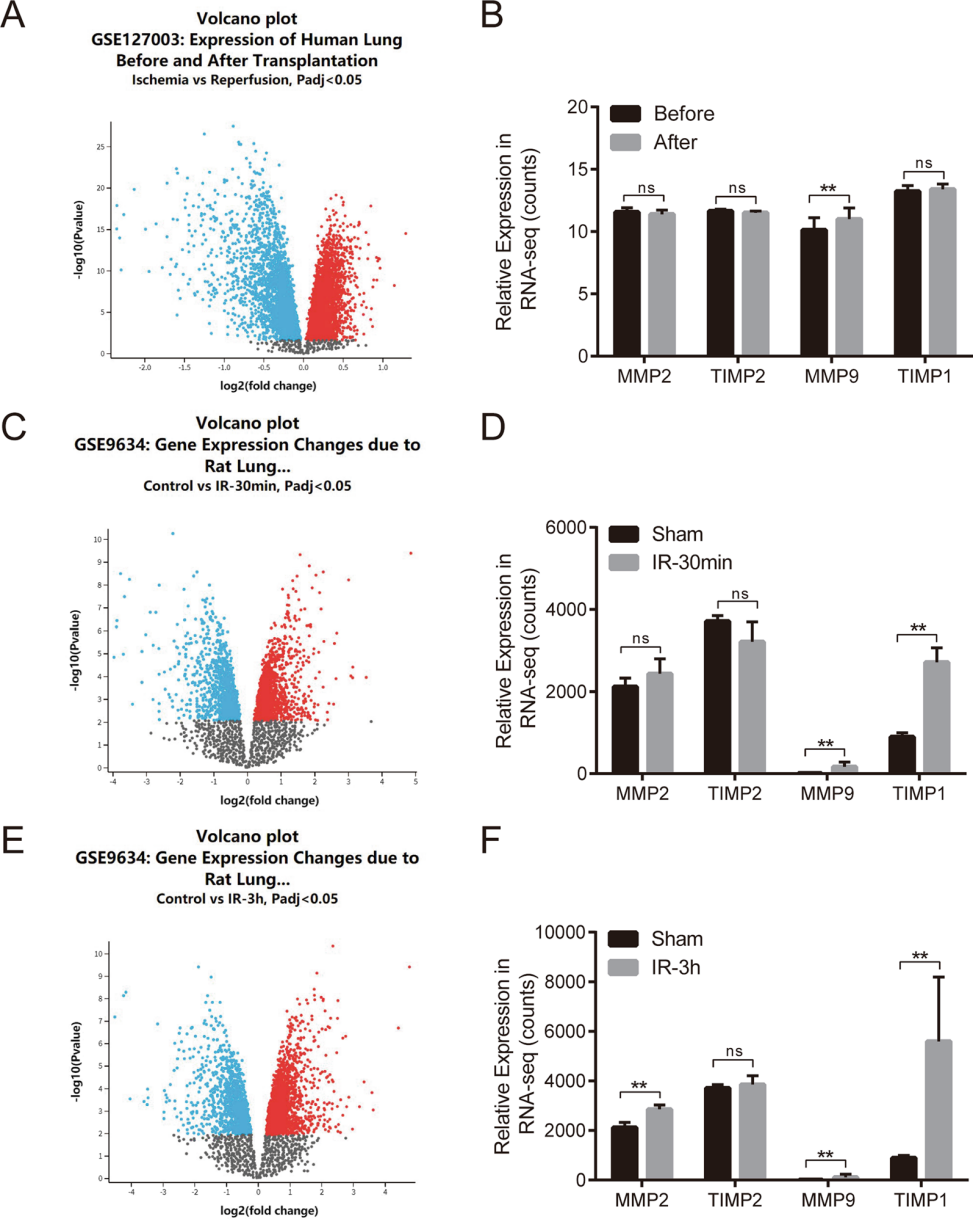
GEO database shows increased expression of MMP2 and MMP9 after LIRI. **a** Volcano plot showing changes in gene expression before and after human lung transplantation in the GEO database. **b** RNA-Seq results showing changes in the expression of MMP2, TIMP2, MMP9, and TIMP1 before and after human lung transplantation. **c** Volcano plot showing changes in gene expression after 30 min of ischemia–reperfusion in rat lung in the GEO database. **d** RNA-Seq results showing changes in the expression of MMP2, TIMP2, MMP9, and TIMP1 in rat lungs after 30 min of ischemia–reperfusion. **e** Volcano plot showing changes in gene expression after 3 h of ischemia–reperfusion in rat lungs from the GEO database. **f** RNA-Seq results showing changes in the expression of MMP2, TIMP2, MMP9, and TIMP1 in rat lungs after 3 h of ischemia–reperfusion

To initially explore the relationship of MMP2 and MMP9 with lung ischemia–reperfusion injury, we performed relevant bioinformatics analysis using the GEO database. From the human lung transplantation RNA-Seq data [35], 2601 genes were observed to be differentially expressed (Fig. 1A). The expression of MMP9 was significantly increased (Fig. 1B). Similarly, the rat lung ischemia–reperfusion RNA-Seq data showed many differentially expressed genes [36], both after IR-30 min (Fig. 1C) and IR-3 h (Fig. 1E). Further analysis showed that the expression of MMP9 increased after IR-30 min (Fig. 1D), but expression of both MMP2 and MMP9 increased after IR-3 h (Fig. 1F).

### Expression of MMP2 and MMP9 is increased in the LIRI mouse model

**Fig. 2:**
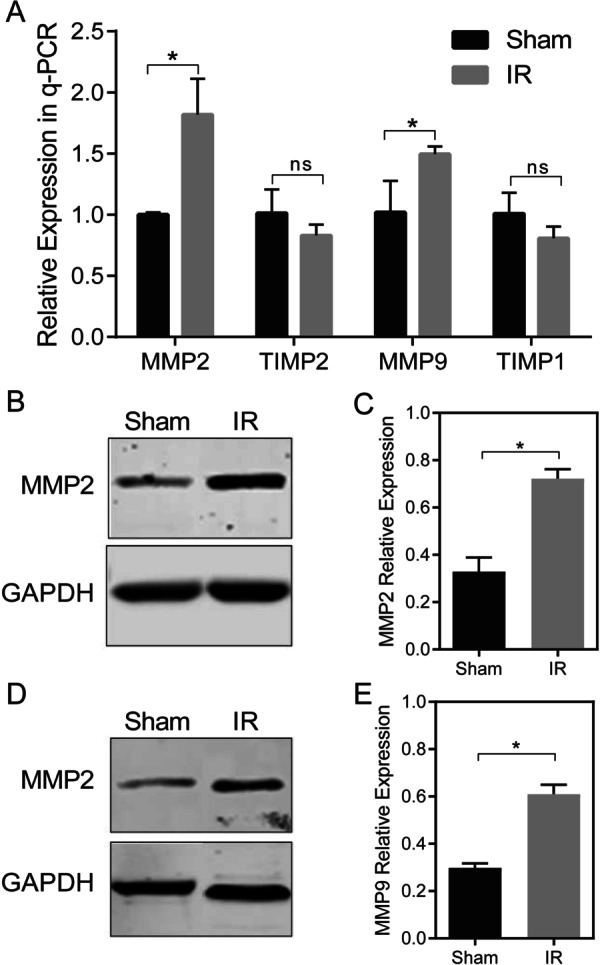
Increase MMP2 and MMP9 expression following LIRI in the mouse model. **a** qPCR results of MMP2, TIMP2, MMP9, and TIMP1 in lung tissues of IR group versus sham group. **b** Western blot results of MMP2 in lung tissues of IR group versus sham group. **c** Expression of MMP2 in lung tissues of IR group versus sham group. **d** Western blot results of MMP9 in lung tissues of IR group versus sham group. **e** Expression of MMP9 in lung tissues of IR group versus sham group

Though MMP2 and MMP9 mRNA expression was increased, the activity of the MMP2 and MMP9 is indeterminate. We further investigated the changes in protein expression of MMP2 and MMP9 by establishing a mouse model of left lung ischemia–reperfusion. In the IR model, the qPCR results showed increased expression of MMP2 and MMP9 in the IR group compared with the sham group, and TIMP2 and TIMP1 showed no significant difference between the two groups, but showed a decreasing trend (Fig. 2A). The Western blot results showed that the MMP2 and MMP9 protein levels were higher in the IR group compared with the sham group (Fig. 2B–E).

### MMP2 and MMP9 inhibitor SB-3CT attenuates LIRI

**Fig. 3:**
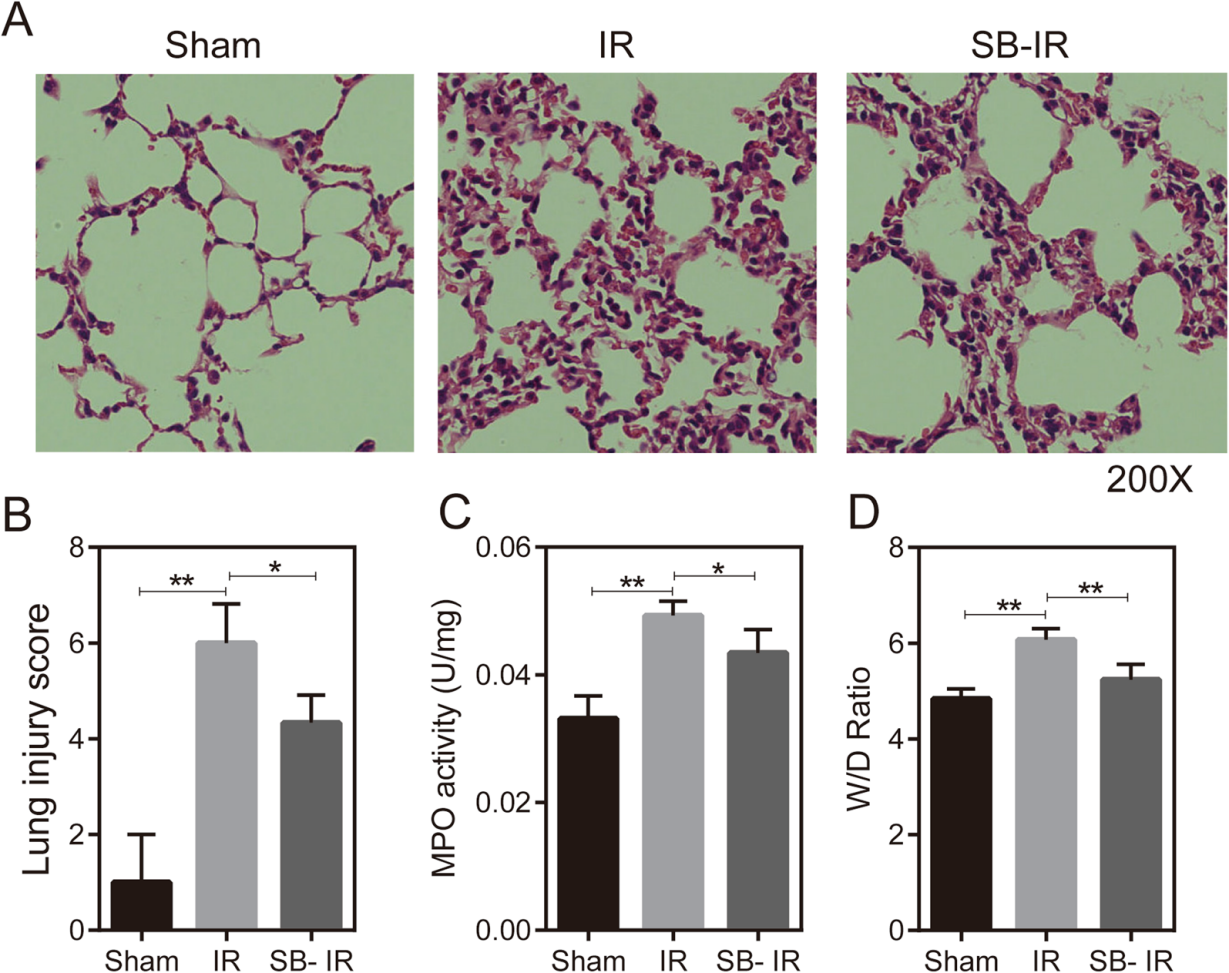
Preconditioning with the MMP2 and MMP9 inhibitor SB-3CT attenuates LIRI severity. **a** H&E staining results (200 ×) of paraffin sections of lung tissue in the sham (n = 5), IR (n = 4), and SB-IR groups (n = 3), **b** Lung injury scores in the sham (n = 5), IR (n = 4), and SB-IR groups (n = 3). **c** MPO activity in the sham (n = 5), IR (n = 4), and SB-IR groups (n = 3). **d** Wet/dry weight ratio of lungs in the sham (n = 5), IR (n = 4), and SB-IR groups (n = 3)

To further verify the role of MMP2 and MMP9 in pulmonary ischemia–reperfusion injury, we collected lung tissues from a mouse lung IR model for H&E staining and assessed the extent of lung injury by pathological injury scoring. Neutrophil infiltration was determined by detection of MPO activity, and the lung wet/dry weight ratio was also measured. In addition, SB-3CT, a specific inhibitor of MMP2 and MMP9, was applied to precondition the mice, which were then subject to lung ischemia–reperfusion, and the corresponding indexes were subsequently examined. H&E staining results showed edema, alveolar septal thickening, and increased cellular infiltration in lung tissue after IR, whereas the corresponding changes were significantly reduced after SB-3CT pretreatment (Fig. 3A), lung pathological damage score increased, MPO activity increased, as did the wet/dry (W/D) ratio after IR, whereas the above changes were alleviated after SB-3CT preconditioning (Fig. 3B–D).

### Elevated levels of pyroptosis due to LIRI in the mouse model

**Fig. 4:**
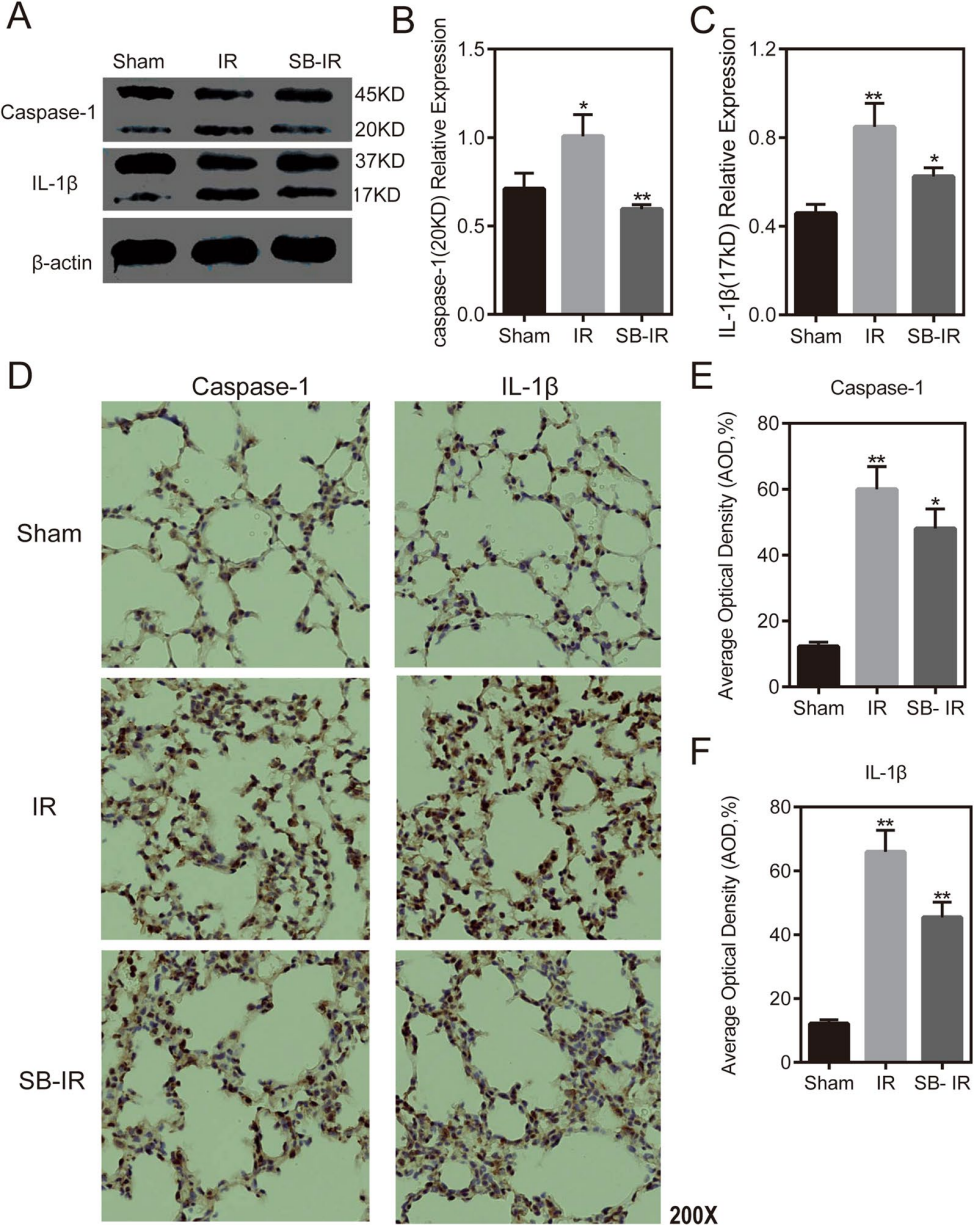
Elevated levels of pyroptosis due to LIRI, and SB-3CT preconditioning leads to reduced levels of lung pyroptosis after LIRI in the mouse model. **a** Western blot results of caspase-1 and IL-1β in the sham, IR, and SB-IR groups. **b** Protein expression of caspase-1 in the sham, IR, and SB-IR groups. **c** Protein expression of IL-1β in the sham, IR, and SB-IR groups. d. IHC results of caspase-1 and IL-1β in the sham, IR, and SB-IR groups. e. Average optical density (AOD) of caspase-1 in the sham, IR, and SB-IR groups. f. AOD of IL-1β in the sham, IR, and SB-IR groups

To examine the changes in the level of pyroptosis after lung ischemia–reperfusion, changes in the expression of caspase-1 and IL-1β were examined in lung tissues of the mouse lung IR model. The results of Western blotting and IHC showed that caspase-1 and IL-1β expression both increased after IR (Fig. 4), suggesting the occurrence of pyroptosis.

### Preconditioning with the MMP2 and MMP9 inhibitor SB-3CT leads to reduced levels of lung pyroptosis following LIRI

The next step was to detect the changes in the pyroptosis index by applying the MMP2 and MMP9 inhibitors to illustrate the effects of MMP2 and MMP9 on cell pyroptosis in LIRI. The results show that pretreatment with SB-3CT resulted in reduced expression levels of caspase-1 and IL-1β after IR (Fig. 4), suggesting that MMP2 and MMP9 play a role in inhibiting cell pyroptosis.

## Discussion

**Fig. 5:**
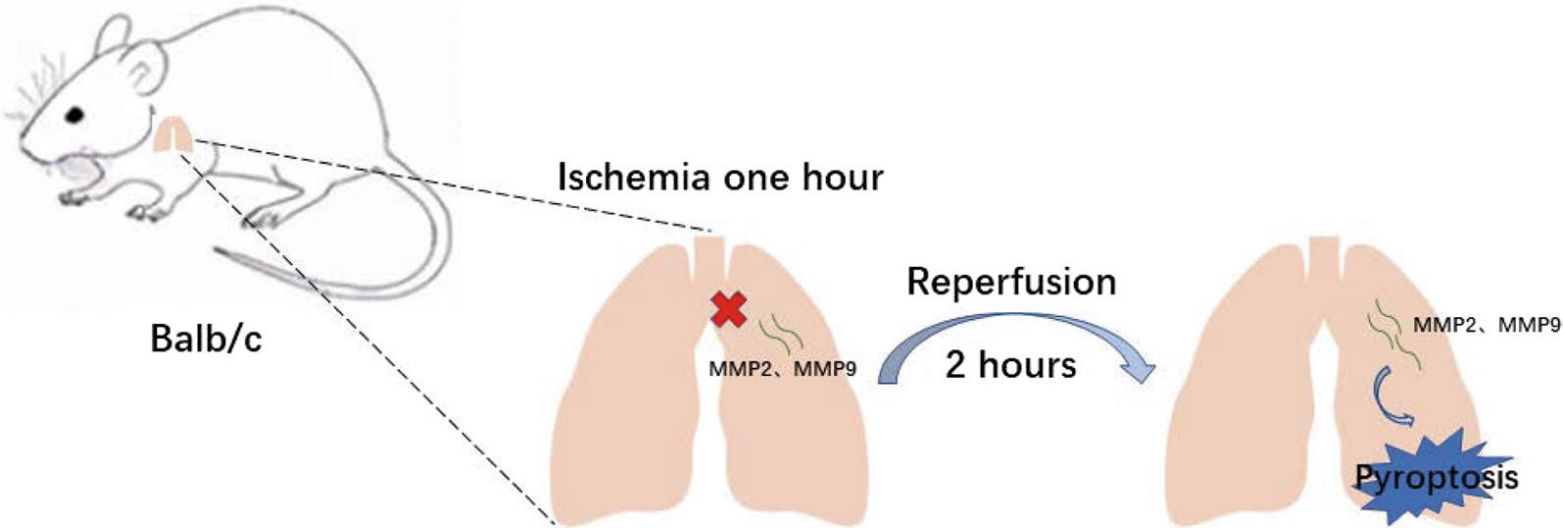
Experimental flow and mechanism diagram

MMPs are a family of zinc-dependent endopeptidases, derived from inactive precursor zymogens, that can degrade almost all extracellular matrix components, except polysaccharides [37]. As two representative members, MMP2 and MMP9 are capable of degrading the extracellular matrix (ECM) component of the alveolar basement membrane [23]. MMPs are inhibited in vivo by natural specific inhibitors, TIMPs, with which they can form complexes [38]. A recent study found that MMP9 mediates hyperglycemia-induced hepatocyte pyroptosis, suggesting a role for MMPs in pyroptosis [39]. Pyroptosis, as one of the methods of programmed cell death, is involved in the development of IRI in several organs. In our experiments, we observed elevated MMP2 and MMP9 expression after lung ischemia–reperfusion at the tissue level in three separate organisms: human, rat, and mouse. While focusing on the changes in MMPs, we also examined the changes in the corresponding TIMPs to more fully illustrate the role of MMP2 and MMP9 in LIRI. We applied a specific inhibitor of MMP2/MMP9 and observed a significant decrease in the indexes related to the extent of LIRI, and a simultaneous decrease in pyroptosis was also observed, suggesting that MMPs may be involved in LIRI by affecting cell pyroptosis; inhibition of their expression can reduce the occurrence of pyroptosis and, thus, extent of LIRI (Fig. 5).

An in vivo study of brain IRI showed that MMPs are involved in the opening of the blood–brain barrier [40]. MMP2 and MMP9 are also found to be overexpressed and activated in experimental lung IRI [23, 41]. One study demonstrated increased MMP2 and MMP9 expression in a lung transplantation model. It was also shown that non-selective inhibition of MMP prevented perivascular gelatinolytic activity, PMN infiltration, and alveolar capillary membrane leakage and improved oxygenation [41]. By contrast, our experiments demonstrated that the specific inhibition of MMP2 and MMP9 expression resulted in reduced lung injury conditions and MPO activity in a LIRI model, suggesting that MMP2 and MMP9 are involved in LIRI by promoting neutrophil infiltration. The infiltration of neutrophils into the pulmonary circulation and lung interstitium can further aggravate the lung injury [42]. Neutrophils can stimulate adherence by producing multiple proinflammatory cytokines and chemo-tactic agents and cause direct pulmonary injury by releasing elastase and other proteases [43].

Tissue inhibitors of metalloproteinases play a key role in regulating MMP activity and ECM formation [44, 45], as an imbalance between MMPs and TIMPs significantly disrupts ECM integrity in a variety of diseases [46, 47]. In the present study, MMP2 and MMP9 expression significantly increased after LIRI, but there was a corresponding decrease in TIMP2 and TIMP1 expression. This indicates that in the context of IR, the original MMPs inhibitors, struggle to fulfill their role in inhibiting MMPs in vivo and require the intervention of external inhibitors.

Pyroptosis often occurs in various organs and tissues that are exposed to stressful environments; for example, studies show that pyroptosis plays an important role in myocardial IRI [48] and is also thought to play a key role in IRI of the retina [49]. Experiments also demonstrate that the activation of pyroptosis is an important feature of acute lung injury induced by renal ischemia–reperfusion [50]. Our experimental results showed increased expression of caspase-1 and IL-1β after lung ischemia–reperfusion, suggesting the occurrence of pyroptosis, which was confirmed by immunohistochemical staining. The expression of MMP2 and MMP9 showed simultaneous changes with cell scoring indicators. Caspase-1 and IL-1β expression decreased after the application of an MMP-specific inhibitor, suggesting that MMP2 and MMP9 inhibit pyroptosis after pulmonary ischemia–reperfusion. Studies previously showed that MMP9 can regulate inflammation [51] and may be involved in cellular pyroptosis by promoting IL-1β release [52].

SB-3CT, a highly selective inhibitor, is known to specifically target only MMP2 and MMP9. Previous studies show that SB-3CT protects against cerebral IRI. The ability of SB-3CT to inhibit MMP9 activity in vivo maintains laminin integrity, antagonizes the contraction and loss of outer membrane cells, and preserves laminin-positive staining of outer membrane cells and endothelial cells [53]. In this experiment, we verified the inhibitory effect of SB-3CT on MMP2 and MMP9 activity, which thereby reduced LIRI, inflammatory factor secretion, and neutrophil aggregation. We also found a reduction in pyroptosis after inhibitor application, suggesting that MMPs may promote the progression of IRI by affecting pyroptosis and cell scoring, providing a new idea and target for research into related treatment. The results also suggest that MMPs may contribute to the progression of ischemia–reperfusion injury by affecting cell scoring, providing new research ideas and targets for related treatments.

Our study also has some limitations: the causal relationship between pyroptosis and LIRI needs to be investigated, and further experiments are needed to verify the specific signaling pathways between MMP2, MMP9, and pyroptosis. Considering the diversity and complexity of cells involved in IRI in lung tissue, we did not perform relevant cytological experiments for resolving the aforementioned issues. We are in the process of identifying the cell types to be studied next and further mechanisms of action through different pre-experiments. LIRI models cannot fully mimic the pathophysiological changes during lung transplantation, and lung transplantation models or clinical lung transplantation specimens are still needed to further validate our results, especially the role of MMP2.

## Conclusions

Our experiments demonstrate that SB-3CT can reduce the alveolar injury and neutrophil infiltration induced by IR in lungs by inhibiting the expression of MMP2 and MMP9 and affecting their balance with the corresponding TIMPs, simultaneously reducing the extent of pyroptosis during this process, suggesting that MMP2 and MMP9 may promote the occurrence of LIRI by promoting pyroptosis.

## Supplementary Information

**Additional file 1:** The dataset of GSE9634 which is recorded the rat lung ischemia–reperfusion tissue RNA-Seq data after IR-3h, in the GEO database. (Figure 1E)

**Additional file 2:** The dataset of GSE9634 which is recorded the rat lung ischemia–reperfusion tissue RNA-Seq data after IR-30min, in the GEO database. (Figure 1C)

**Additional file 3:** The dataset of GSE127003 which is recorded before and after human lung transplantation RNA-Seq data of lung tissue, in the GEO database. (Figure 1A)

**Additional file 4:** Western blot images raw data of Figure 2B

**Additional file 5:** Western blot images raw data of Figure 4A

**Additional file 6:** The representative image of H&E staining paraffin sections of lung tissue in the sham, IR and SB-IR group separately. (Figure 3A)

**Additional file 7:** The representative image of caspase-1 and IL-1β expression in sham, IR and SB-IR group by immunohistochemistry separately. (Figure 4D)

**Additional file 8:** Lung injury score data. (Figure 3B)

**Additional file 9:** The sample Cq value in RT-qPCR. (Figure 2A)

## Data Availability

All data relevant to the study are included in the article or uploaded as supporting information.

## Abbreviations

PGD: Primary graft dysfunction
LIRI: Lung ischemia–reperfusion injury
MMPs: Matrix metalloproteinases
PMN: Neutrophil
IR-SB: SB-3CT pretreatment group
MPO: Myeloperoxidase
W/D: Wet-to-dry weight ratio
