# Supplementary material for: MMP2 and MMP9 contribute to lung ischemia–reperfusion injury via promoting pyroptosis in mice
Source: BMC Pulm Med. 2022 Jun 15;22:230. doi: 10.1186/s12890-022-02018-7 (PMC9202153; doi:10.1186/s12890-022-02018-7)

Western blot images of Figure 2B. The blots were cut prior to hybridisation with antibodies during blotting. The images on the left (A) shows all blots and replicates of MMP2 and GAPDH, on the right (B) shows the MMP9 and GAPDH. The inner red rectangle are the representative blots.

A

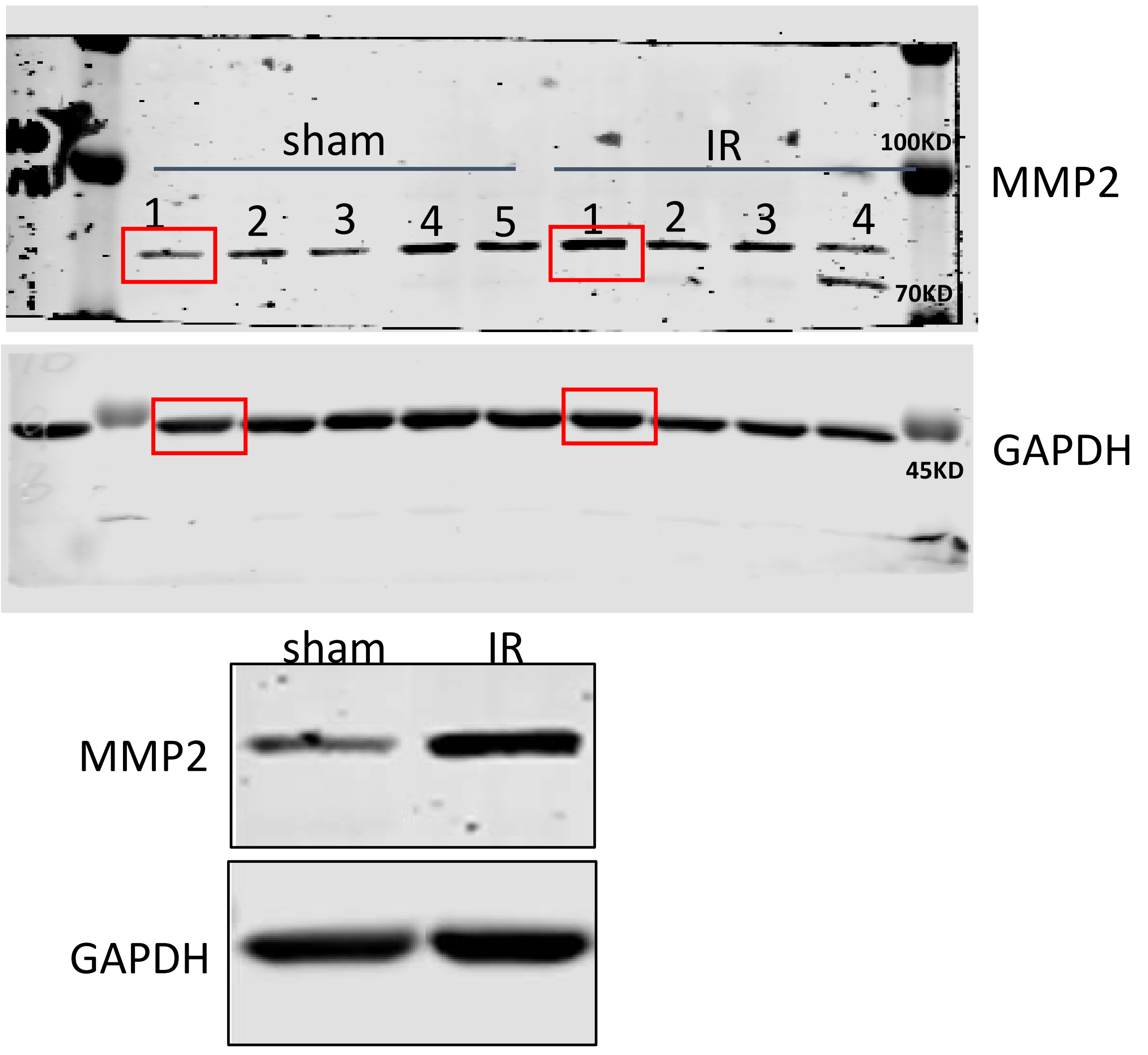

B

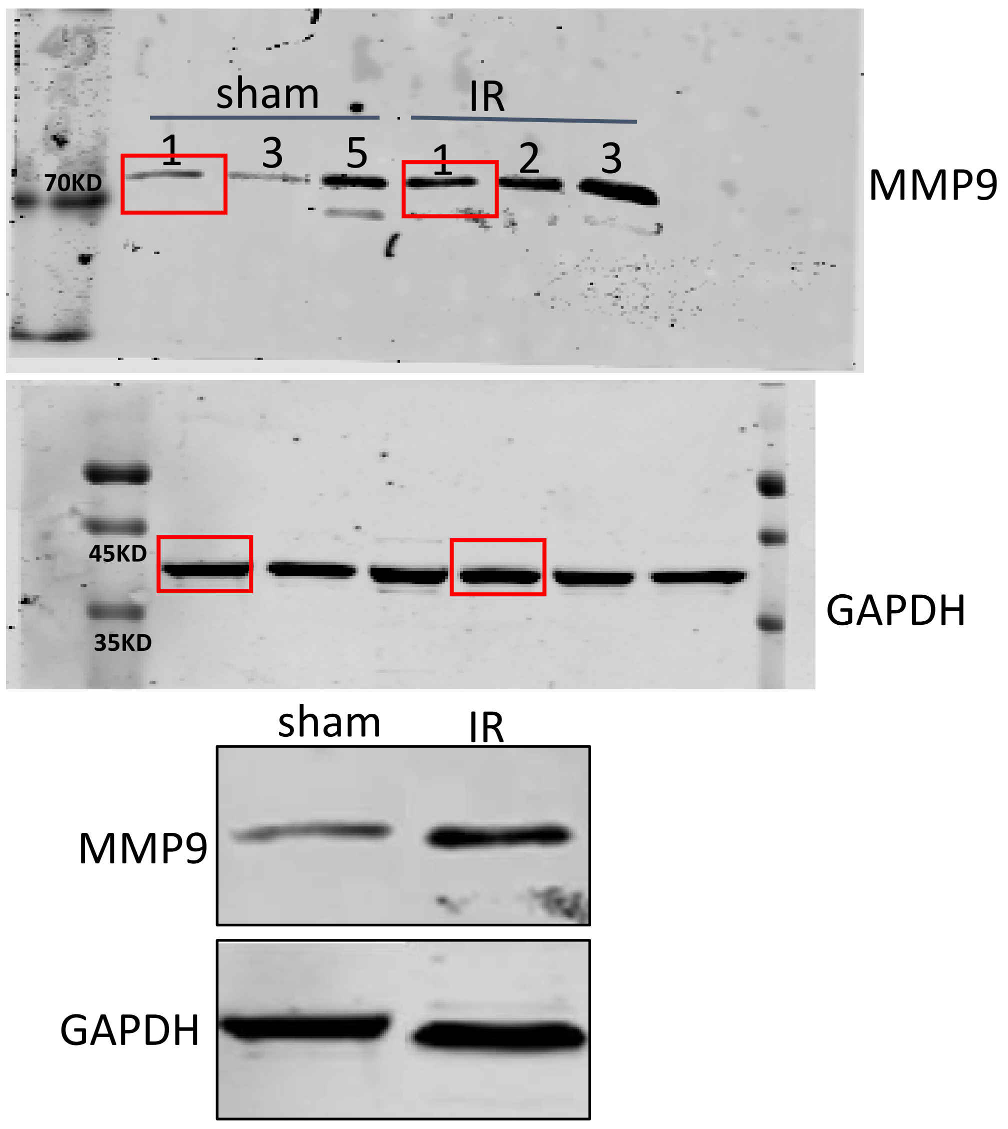

Supplement: Supplementary file 4 — Additional file 4: Western blot images raw data of Figure 2B [file 12890_2022_2018_MOESM4_ESM.pdf]
