# Supplementary material for: MMP2 and MMP9 contribute to lung ischemia–reperfusion injury via promoting pyroptosis in mice
Source: BMC Pulm Med. 2022 Jun 15;22:230. doi: 10.1186/s12890-022-02018-7 (PMC9202153; doi:10.1186/s12890-022-02018-7)

Western blot images of Figure 4A. The blots were cut prior to hybridisation with antibodies during blotting. The images show all blots and replicates of caspase-1, IL-1β, and β-actin. The inner red rectangle are the representative blots.

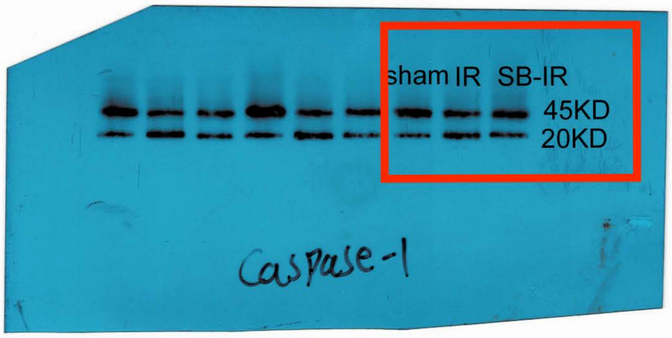

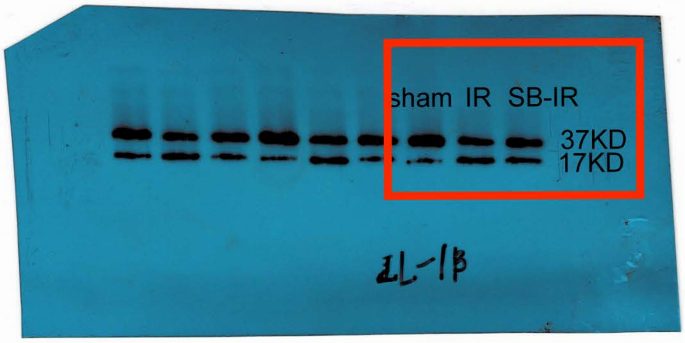

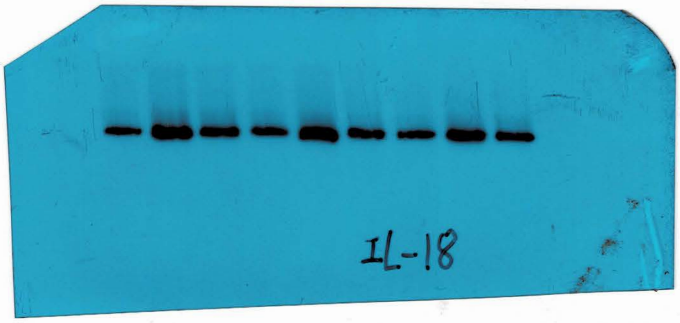

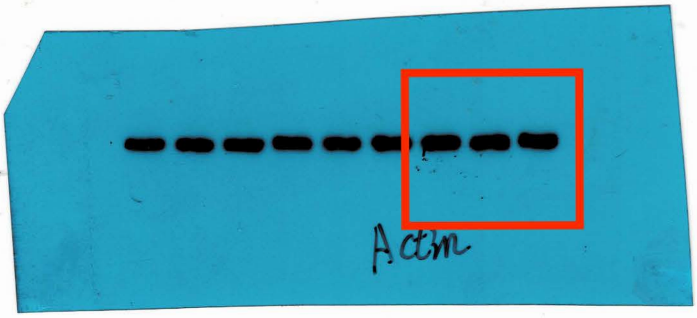

Supplement: Supplementary file 5 — Additional file 5: Western blot images raw data of Figure 4A [file 12890_2022_2018_MOESM5_ESM.pdf]
