# Supplementary material for: MMP2 and MMP9 contribute to lung ischemia–reperfusion injury via promoting pyroptosis in mice
Source: BMC Pulm Med. 2022 Jun 15;22:230. doi: 10.1186/s12890-022-02018-7 (PMC9202153; doi:10.1186/s12890-022-02018-7)

The representative image of H&E staining paraffin sections of lung tissue in the sham group

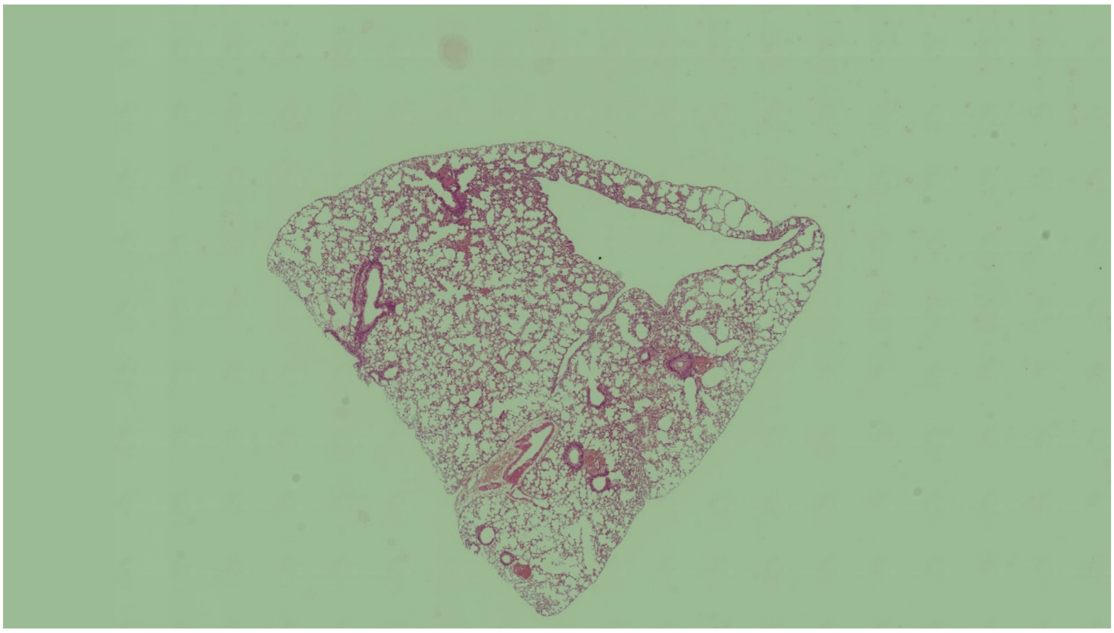

The representative image of H&E staining paraffin sections of lung tissue in the IR group

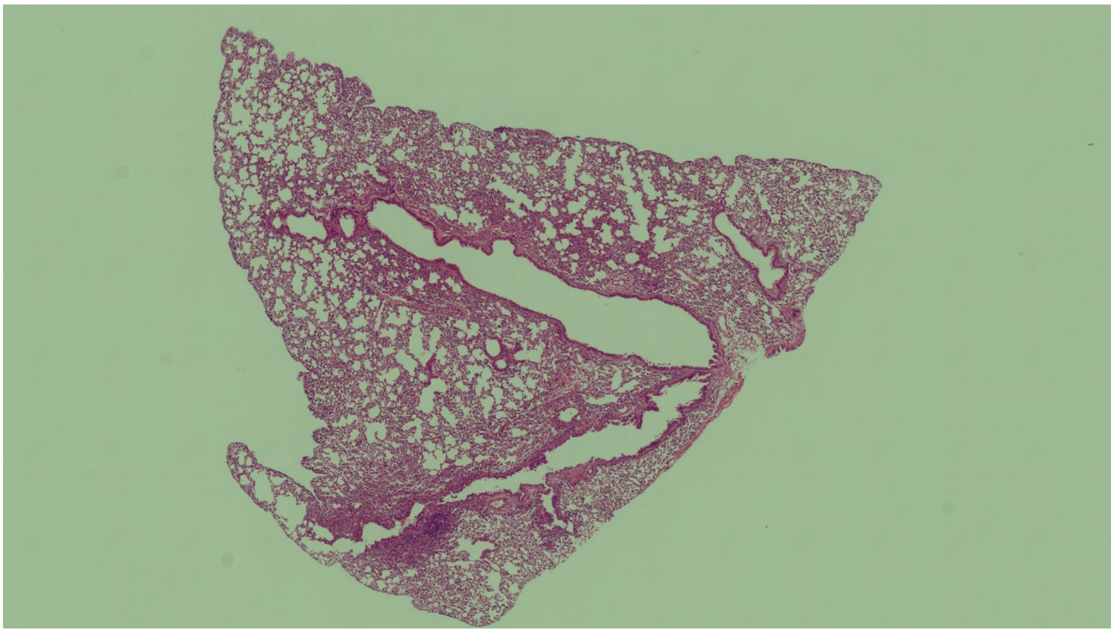

The representative image of H&E staining paraffin sections of lung tissue in the SB-IR group

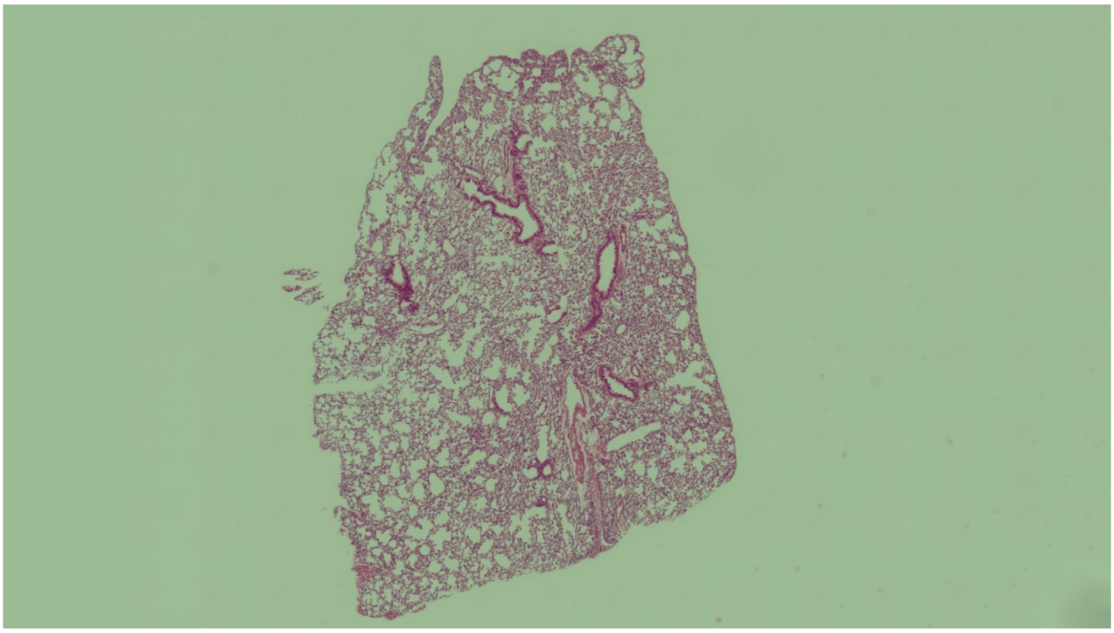

Supplement: Supplementary file 6 — Additional file 6: The representative image of H&E staining paraffin sections of lung tissue in the sham, IR and SB-IR group separately. (Figure 3A) [file 12890_2022_2018_MOESM6_ESM.pdf]
