# Supplementary material for: MMP2 and MMP9 contribute to lung ischemia–reperfusion injury via promoting pyroptosis in mice
Source: BMC Pulm Med. 2022 Jun 15;22:230. doi: 10.1186/s12890-022-02018-7 (PMC9202153; doi:10.1186/s12890-022-02018-7)

The representative image of caspase-1 expression in sham group by immunohistochemistry

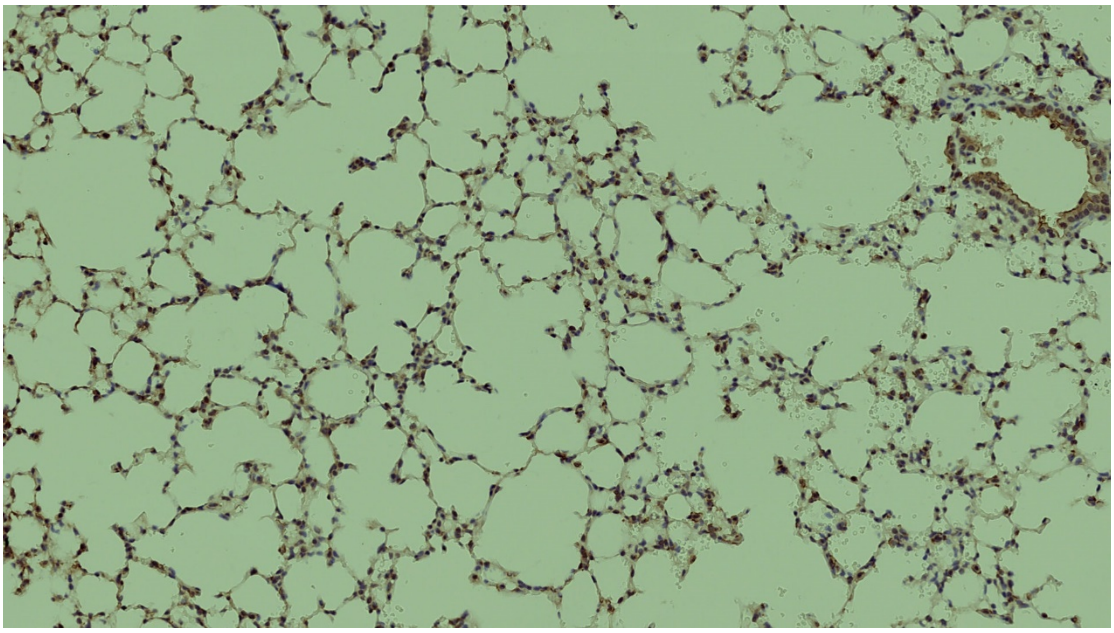

The representative image of caspase-1 expression in IR group by immunohistochemistry

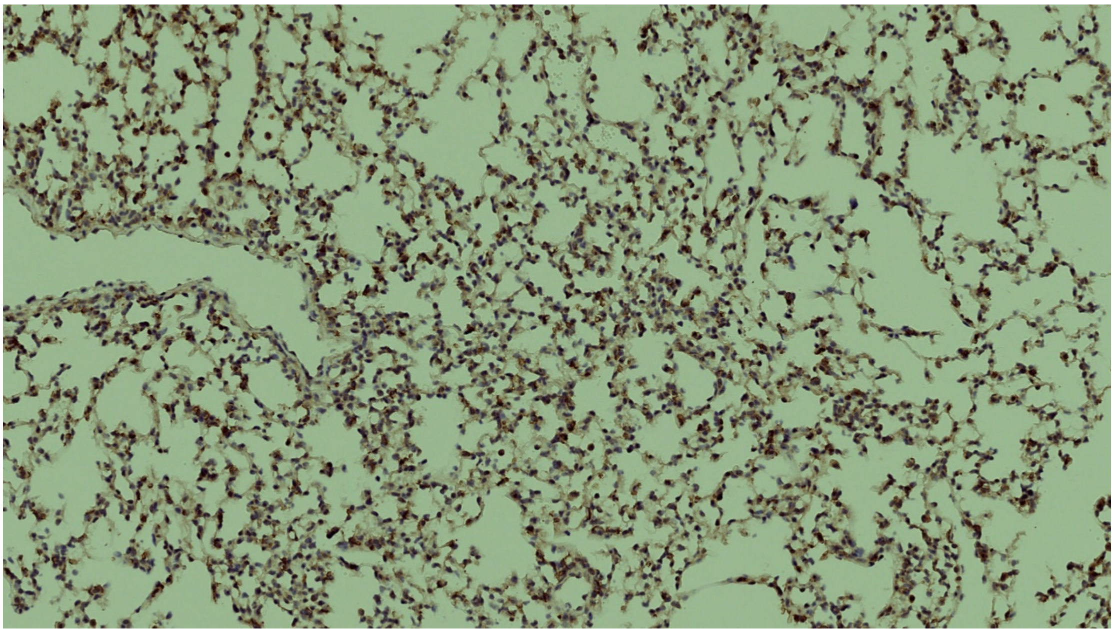

The representative image of caspase-1 expression in SB-IR group by immunohistochemistry

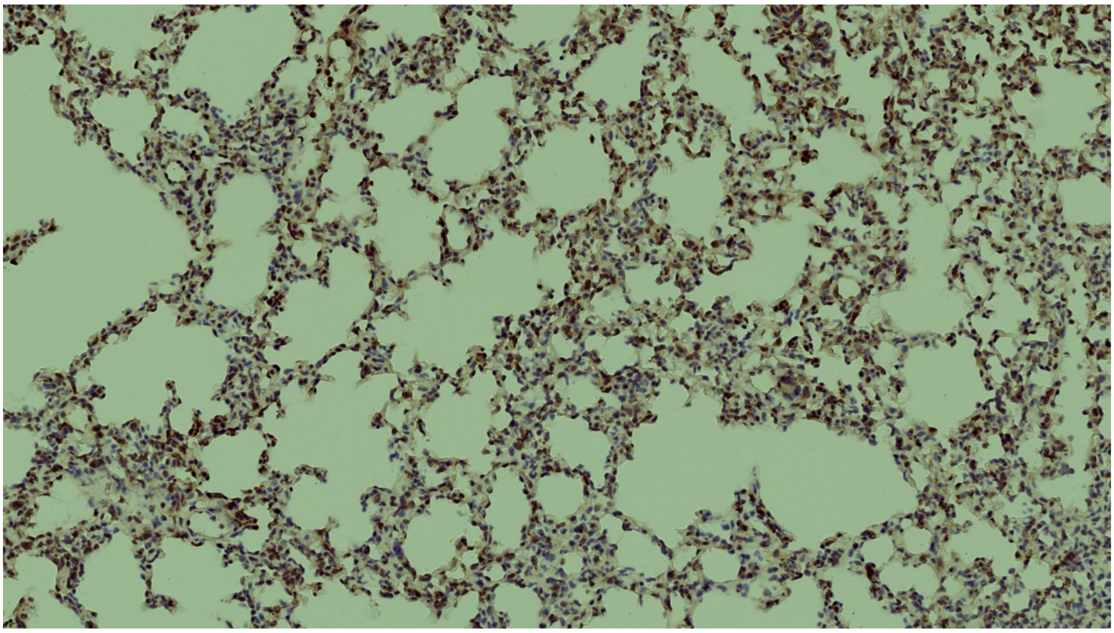

The representative image of IL-1β expression in sham group by immunohistochemistry

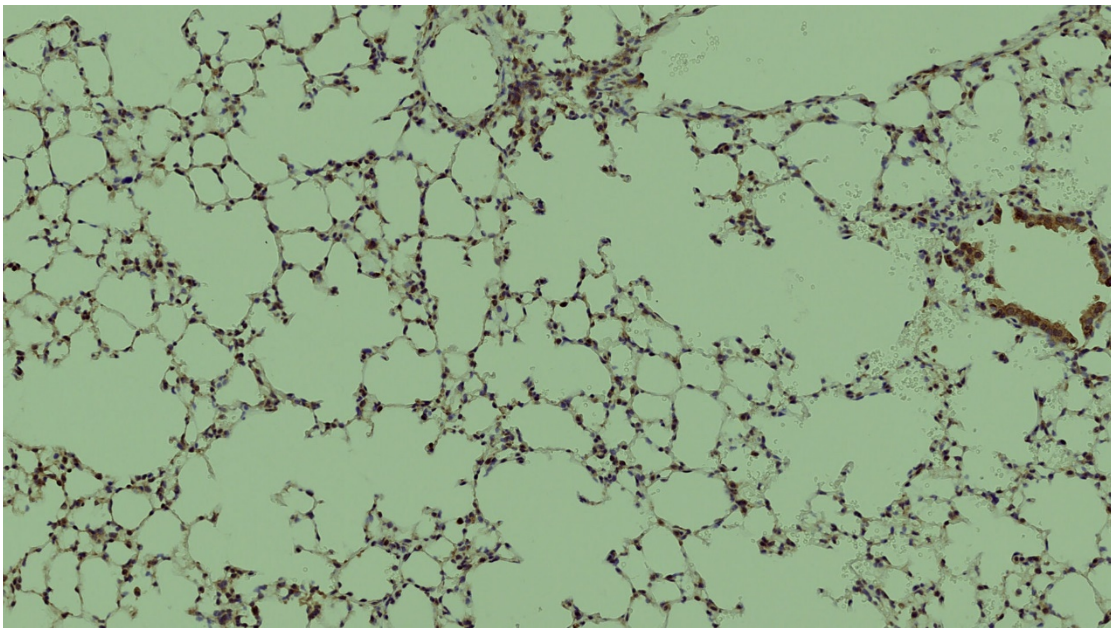

The representative image of IL-1β expression in IR group by immunohistochemistry

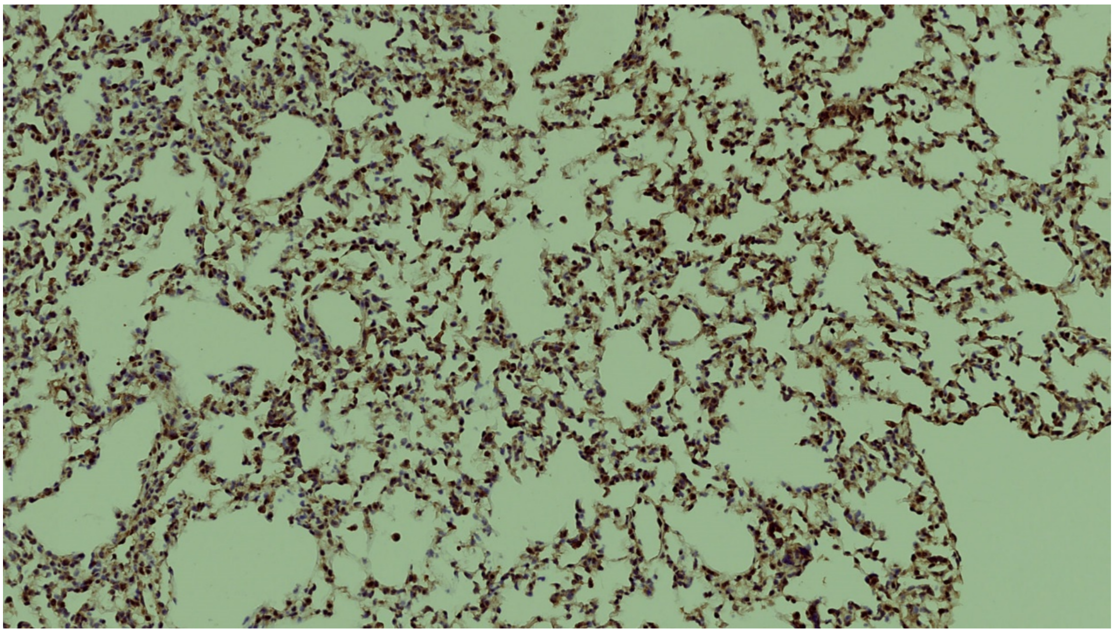

The representative image of IL-1β expression in SB-IR group by immunohistochemistry

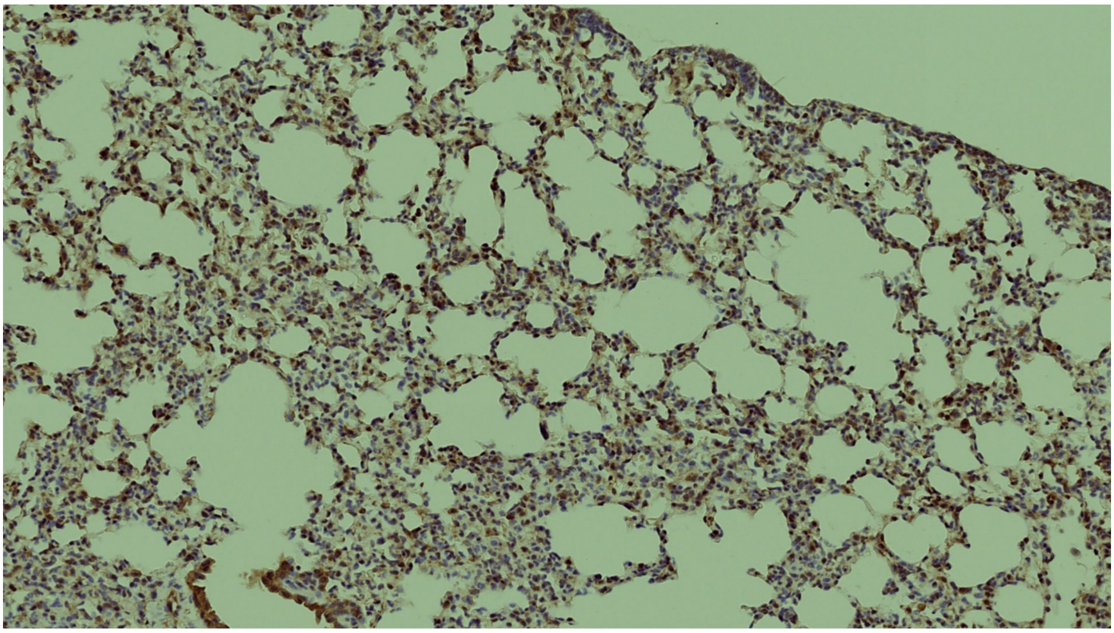

Supplement: Supplementary file 7 — Additional file 7: The representative image of caspase-1 and IL-1β expression in sham, IR and SB-IR group by immunohistochemistry separately. (Figure 4D) [file 12890_2022_2018_MOESM7_ESM.pdf]
